# Supplementary material for: Versatile mapping-by-sequencing with Easymap v.2
Source: Front Plant Sci. 2023 Jan 26;14:1042913. doi: 10.3389/fpls.2023.1042913 (PMC9909543; doi:10.3389/fpls.2023.1042913)
Supplement: Supplementary file 1 [file DataSheet_1.zip › Supplementary File 2.PDF]

Versatile mapping-by-sequencing with Easymap v.2

Samuel Daniel Lup, Carla Navarro-Quiles, and José Luis Micol

Instituto de Bioingeniería, Universidad Miguel Hernández, Campus de Elche, 03202 Elche, Spain

Supplementary File S2: Easymap v.2 Quickstart Installation Guide

Easymap v.2 Quickstart Installation Guide

This is a simplified guide for **Easymap v.2** installation in the main supported Operating Systems. Check the **Easymap v.2 Documentation** at http://genetics.edu.umh.es/resources/easymap/ for more detailed information and other installation setups. **System administrator privileges and internet access** are required for the installation. The following steps are meant to simplify the installation; advanced users can alter the process for specific needs.

1 Installation Environment

Easymap v.2 is meant to be installed within a **Linux system**, these are a few setups that can be used:

- A physical machine running **Ubuntu**, **Linux AMI** or **Red Hat**
- The **Ubuntu 18.04 app**, available for download in the **Windows 10 Microsoft Store**. *You may be prompted to enable the "Windows Subsystem for Linux" feature of the Windows 10 OS. To do so, run the Windows PowerShell as administrator, run the following command and restart your system:*

```
Enable-WindowsOptionalFeature -Online -FeatureName Microsoft-Windows-Subsystem-Linux
```

- A virtual machine (VM) running Linux within **Windows** or **Mac OS**. *Get the Ubuntu ISO at https://releases.ubuntu.com/18.04.4/ubuntu-18.04.4-live-server-amd64.iso and mount it using Oracle Virtualbox. Performance in virtual machines is limited but can be sufficient. Choose a "Bridged connection" while setting up the virtual machine to be able to access the graphic interface later.*

2 Easymap v.2 Installation

Open the Linux console and run the following commands (ignoring $ symbol):

```
$ cd ~                                                          Moves to home folder

$ wget http://genetics.umh.es/other_files/genetica%20umh%20es   Downloads the Easymap
       /Easymap/easymap-installer-v2.sh                         v.2 installer

$ sudo bash easymap-installer-v2.sh                             Installs Easymap v.2
```

Select your operating system from the interactive menu and wait for the installer to finish. You will get a "Easymap successfully installed" message at the end. Installation can take up to 30 minutes. *In the unlikely case your machine doesn't have wget installed, use the command "`sudo apt install wget`" in Ubuntu or "`sudo yum install wget`" in other distributions.*

3 Access Easymap v.2

- For **local** access, point your web browser to **http://localhost:8100**
- For **remote** access (from another machine or VM), point your web browser to **http://<IP-address>:8100**

Additional notes

- See Easymap v.2 Documentation for further details and other installation setups.
- Manual server rebooting may occasionally be necessary. If at any time you cant access the graphic interface please run the following commands in the console:

```
$ cd ~/easymap.v2                        Moves to easymap v.2 directory

$ bash launch-server.sh <port-number>    Launches easymap v.2 server
```

Important: You must replace `<port-number>` with any number "81XX" between 8101 and 8200 and use the same port number to access easymap (http://localhost:81XX).

- A preview version of the graphical interface is available for testing at: http://atlas.umh.es/easymapv2
