# Supplementary material for: Versatile mapping-by-sequencing with Easymap v.2
Source: Front Plant Sci. 2023 Jan 26;14:1042913. doi: 10.3389/fpls.2023.1042913 (PMC9909543; doi:10.3389/fpls.2023.1042913)
Supplement: Supplementary file 2 [file Table_1.pdf]

Supplementary Table S1. Validation of the Easymap v.2 variant density mapping workflow with experimental data

Original study	Data source	Species	Mapping strategy	Mutant	Variants identified by Easymap v.2	Project name in the preview Easymap v.2 interface
Zuryn *et al*. (2010)	Provided by the authors	*C. elegans*	Backcross	*mutA*	The causal mutation and two other nonsynonymous mutations in the candidate region	2020-08-27-13:19:26_mutA
				mutD	The correct candidate region (the causal mutation was unknown)	2020-08-27-17:07:57_mutD
				mutH	The correct candidate region (the causal mutation was unknown)	2020-08-27-20:56:44_mutH
Zuryn *et al*. (2014)	Provided by the authors	*C. elegans*	Backcross	*jmjd-3.1 (fp25)*	The causal mutation was the only nonsynonymous mutation in the candidate region	2020-08-29-18:34:13_37
				egl-27	The causal mutation and two other nonsynonymous mutations in the candidate region	2020-08-28-20:38:40_83
Svensk *et al*. (2016)	Provided by the authors	*C. elegans*	Backcross	*sma-1*	The causal mutation and eight other nonsynonymous mutations in the candidate region	2020-09-08-14:04:40_40
				dpy-23	The causal mutation and ten other nonsynonymous mutations in the candidate region	2020-09-08-20:59:14_41
				et43	The causal mutation and another nonsynonymous mutation in three candidate regions	2020-09-09-09:07:16_43
Klein *et al*. (2018)*	PRJNA476333	*Z. mays*	Outcross	*ten*	The causal mutation and 249 other nonsynonymous mutations in the candidate region	2020-08-27-10:08:18_TEN

The reference genome and annotation files of *C. elegans* and *Z. mays* were downloaded from the Assembly database of the National Center for Biotechnology Information (NCBI; https://www.ncbi.nlm.nih.gov/assembly/) under accessions WBcel235 and B73 RefGen_v4, respectively. The data was obtained from the Sequence Read Archive (SRA) of the NCBI (https://www.ncbi.nlm.nih.gov/sra). *The mapping population of this dataset was obtained by a single outcross to the reference strain.
