# Supplementary material for: Versatile mapping-by-sequencing with Easymap v.2
Source: Front Plant Sci. 2023 Jan 26;14:1042913. doi: 10.3389/fpls.2023.1042913 (PMC9909543; doi:10.3389/fpls.2023.1042913)
Supplement: Supplementary file 3 [file Table_2.pdf]

Supplementary Table S2. Validation of the Easymap v.2 QTL-seq workflow with experimental data

Original study	Data source	Species, cultivar (cv.)	MP[1]	Trait, mutation or QTL	Results obtained by Easymap v.2	Project name in the preview Easymap v.2 interface
Illa-Berenguer *et al*. (2015)	Provided by the authors	*S. lycopersicum*	F_2	*fw11.2*	The QTL was properly mapped*	2021-11-06-11:59:05_139
			F_2	*lcn2.4*, *fw3.3*, *lcn5.1*, *lcn6.1*	The two major QTL were detected, and two minor QTL were detected by visual inspection of the report*	2021-11-13-17:39:12_75
			F_2	*fw1.1*	A narrower QTL was defined within the previously published QTL	2021-11-06-11:59:19_143
Fekih *et al*. (2013)	DRA001007	*O. sativa*, cv. Hitomebore	M_3	Hit11440	The QTL was not properly mapped due to low SNP density but was detected by visual inspection of the report	2021-11-06-11:59:30_Hit11440
			M_3	Hit9188	The QTL was properly mapped	2021-11-06-11:59:35_Hit9188
Hisano *et al*. (2017)	PRJDB4643	*H. vulgare*	DH[2]	*blp*	The QTL was properly mapped, and the causal mutation was detected	2021-11-06-11:59:25_blp
			DH	Net blotch resistance	A narrower QTL was defined within the previously published QTL	2021-11-06-11:59:40_nbr
Takagi *et al*. (2013)	DRA000809	*O. sativa*, cv. Dunghan Shali	F_2	Seedling vigor	The major QTL was detected, and the second QTL was visible*	2021-11-06-11:59:54_OsDung
		O. sativa, cv. Nortai	RIL	Blast fungus resistance	The QTL was properly mapped with a wider region of interest	2021-11-06-12:00:04_OsNor
		O. sativa, cv. Arroz da terra	RIL	Germination at low temperatures	Two QTL were properly mapped, and the third QTL was manually detected by visual inspection of the report*	2021-11-06-11:59:48_OsArroz
		O. sativa, cv. Iwate96	RIL	Grain amylose content	The QTL was properly mapped*	2021-11-06-11:59:56_OsIwa
Wang *et al*. (2018)	SRR6831103, SRR6831101	*O. sativa*, cv. Nipponbare	F_2	*wb1*	The QTL was properly mapped, and the causal mutation was detected	2021-11-06-12:00:40_Wang18
Yang *et al*. (2017)	SRR5739122, SRR5739123	*O. sativa*, cv. Nipponbare	F_2	*qNUE6*	The QTL was properly mapped	2021-11-30-18:17:01_Yang2017

The reference genome and annotation files of *S. lycopersicum* (accession SL2.50), *O. sativa* cv. Hitomebore (Osat_hitom_01) and *O. sativa* cv. Nipponbare (IRGSP-1.0) were downloaded from the Assembly database of the NCBI. The reference genome and annotation files of *H. vulgare* (MorexV3_pseudomolecules_assembl) were downloaded from Ensembl Plants (http://plants.ensembl.org/index.html). The data was obtained from SRA. [1]Mapping population. [2]Double haploid. *Read depths < 8×.
